# Where will I end up? Relative location of sports medicine fellowship compared to residency among recent graduates

**DOI:** 10.1186/s12909-026-08573-5

**Published:** 2026-01-12

**Authors:** Jimmy Wen, David Park, Daniel Razick, Mouhamad Shehabat, Burhaan Syed, Muzammil Akhtar, Bhagvat Maheta, Caroline Goswami, Naomi Pai, Apple Zhu, Jose Luis Puglisi, Sarah Preiss-Farzanegan

**Affiliations:** 1 College of Medicine, California Northstate University, 9700 W Taron Dr, Elk Grove, CA 95757 USA; 2 Department of Surgery, Northwestern Memorial Hospital, 676 N. St. Clair St., Suite 2320, Chicago, IL 60611 USA; 3 Department of Internal Medicine, California Pacific Medical Center, CA 1101 Van Ness Ave, 94109 San Francisco, USA; 4 University of California, Berkeley, 110 Sproul Hall #5800, Berkeley, CA 94720 USA

**Keywords:** Sports medicine, Fellowship training, Graduate medical education, Education and/Or curriculum development, Advising students and residents

## Abstract

**Background:**

Sports Medicine (SM) fellowships have garnered increased interest over the past several decades as greater emphasis has been placed on physical activity as a cornerstone of well-being. Fellows usually complete a residency in Family Medicine (FM), Physical Medicine & Rehabilitation (PM&R), Pediatrics, Emergency Medicine (EM), or Internal Medicine (IM). This study analyzes the recent geographic trends of SM fellows from 2019 to 2023.

**Methods:**

SM fellows who graduated between 2019 and 2023 were identified from publicly available data, and the distances between SM fellowship and residency were examined. These were categorized as within 100 miles, same state, same region, or different region. Odds ratios (OR) were calculated for the relative locations of a graduate’s primary specialty.

**Results:**

A total of 852 fellows were included. A majority of fellows (64.4%) were from FM, 14.9% from PM&R, 7.1% from Pediatrics, 7% from EM, and 6.2% from IM. FM residency graduates were more likely to stay within 100 miles (OR 1.47), same state (OR: 1.81), or same region (OR: 1.61) (*p* = 0.0093, *p* < 0.0001, *p* = 0.0018, respectively). In contrast, EM residency graduates were more likely not to remain within 100 miles (OR: 0.47) or the same state (OR: 0.52) (*p* = 0.0131, *p* = 0.0214, respectively). Fellows with an FM background were more likely to stay within 100 miles and within the state they trained in, while PM&R graduates were more likely to move greater than 100 miles and to a new state.

**Conclusion:**

Overall, graduates were most likely to stay in the same region for their SM fellowship.

## Background

Sports medicine (SM) is a comprehensive field that extends beyond the confines of traditional medical practice, serving as the intersection between primary care, orthopedics, physiology, cardiology, nutrition, and rehabilitation. SM is a multidisciplinary field, and SM programs accept applicants from Physical Medicine & Rehabilitation (PM&R), Family Medicine (FM), Internal Medicine (IM), Pediatrics, and Emergency Medicine (EM) [1, 2]. SM fellowships are one year in duration and have become increasingly popular, as seen by the significant increase in match rate and doubling number of accredited programs and positions between 2010 and 2021 [2].

A notable paradigm shift has occurred in recent years within the field of SM due to the American College of Sports Medicine (ACSM) championing the concept of “exercise is medicine”. In 2018, the ACSM published its “Prescription for Health,” stating that 150 min of physical activity per week can prevent chronic diseases, including cardiovascular disease, diabetes mellitus type II, and various cancers [3, 4]. SM has continued to transcend its focus on elite athletes to embrace a wider demographic and promote active lifestyles to enhance general well-being, prevent chronic ailments, and improve quality of life.

As the field of SM continues to grow, its role in preventative care and enhancing athletic performance remains a crucial component of modern healthcare [2, 4]. Thus, the demand for SM physicians will continue to evolve across different settings, including academic centers, hospital systems, and community practices [2, 4]. Despite this growth, there is limited data on the geographical distribution from residency to fellowship and early career practice. Understanding these trends has important implications for SM fellowship program development, recruitment/hiring strategies, and workforce planning, especially as programs/hospitals may aim to find trainees who are more likely to stay within a certain region. In the present study, we seek to evaluate the relative distance of fellowships compared to residency for SM fellows between 2019 and 2023. By analyzing the geographical distribution of SM fellowships in the present study, this data will provide valuable information for medical students and residents to better anticipate possible relocation needs and plan out their future careers in SM.

## Methods

### Study design

This retrospective study, conducted in 2023, analyzed current and graduated fellows who completed an Accreditation Council for Graduate Medical Education (ACGME)-accredited SM Fellowship program in the United States between 2019 and 2023. This time period was chosen to reflect the most recent trends and data. The data was obtained from published information online by each fellowship program regarding residency specialty and location. For several programs, graduation information was not publicly available, and a standardized process was initiated to procure the requisite details. A maximum of three communication attempts via email were conducted, and if no response was received, graduate data from these programs were not included in this study. This study was determined to be exempt via the home Institutional Review Board (IRB).

### Statistical analysis

The relative distance of graduate residency location to fellowship destination was categorized to be within a 100-mile radius, same state, same geographic region, and different geographic region. The 100-mile radius was chosen to signify individuals who remained close to their original training institutions, including hospitals within the city and nearby suburbs. If a fellow completed a fellowship and residency that was within 100 miles but crossed state or regional boundaries, they were categorized as within 100 miles. The closest applicable category was used when more than one category applied to a pairing. Thus, these distance categories were both hierarchical and cumulative. This method was chosen to evaluate geographic proximity rather than mutually exclusive classifications. Geographical regions (West, Midwest, Southwest, Southeast, Northeast) were determined by the National Geographic United States region classification [5].

Odds ratios (OR) were calculated to generate forest plots for each specialty. To determine the OR, the number of fellows specific to each category was calculated. Geographic proximity was determined hierarchically for OR calculations. For the OR analysis, each specialty was compared separately against all the other specialties combined. For example, for FM residency graduates, the exposure group consisted of FM residency graduates, while the reference groups consisted of residency graduates from all other specialties pooled together. Therefore, the OR evaluated the odds of staying within a certain geographical proximity for a given specialty relative to all other specialties. Fisher’s exact test was used to calculate a p-value of 0.05. Thus, p-values less than 0.05 were considered statistically significant. Confidence intervals were calculated via the Baptista-Pike method. Calculations were computed using the GraphPad software (Dotmatics, San Diego, CA).

### Map rendering

Pinpoint location maps of the longitudinal coordinates for both residency and fellowship locations were generated via Datawrapper Maps Service (Datawrapper GmbH, Berlin, Germany). Each pinpoint location represents a resident’s location and was color-coded by geographical location, which mirrored the fellowship maps and displayed the origin of each individual.

## Results

This study included data from 135 of 207 ACGME-accredited SM fellowships, accounting for 65.2% of all SM programs in the United States. The programs excluded from the study had either opted out of data collection or did not respond to communication attempts made to the institution. From the participating programs, 838 fellows from 2019 to 2023 were included, with first job data available for 792 fellows. Geographic distribution of the responding programs was broad and slightly more concentrated in the West and Southeast, with slightly less in the Southwest.

### Program locations

**Fig. 1:**
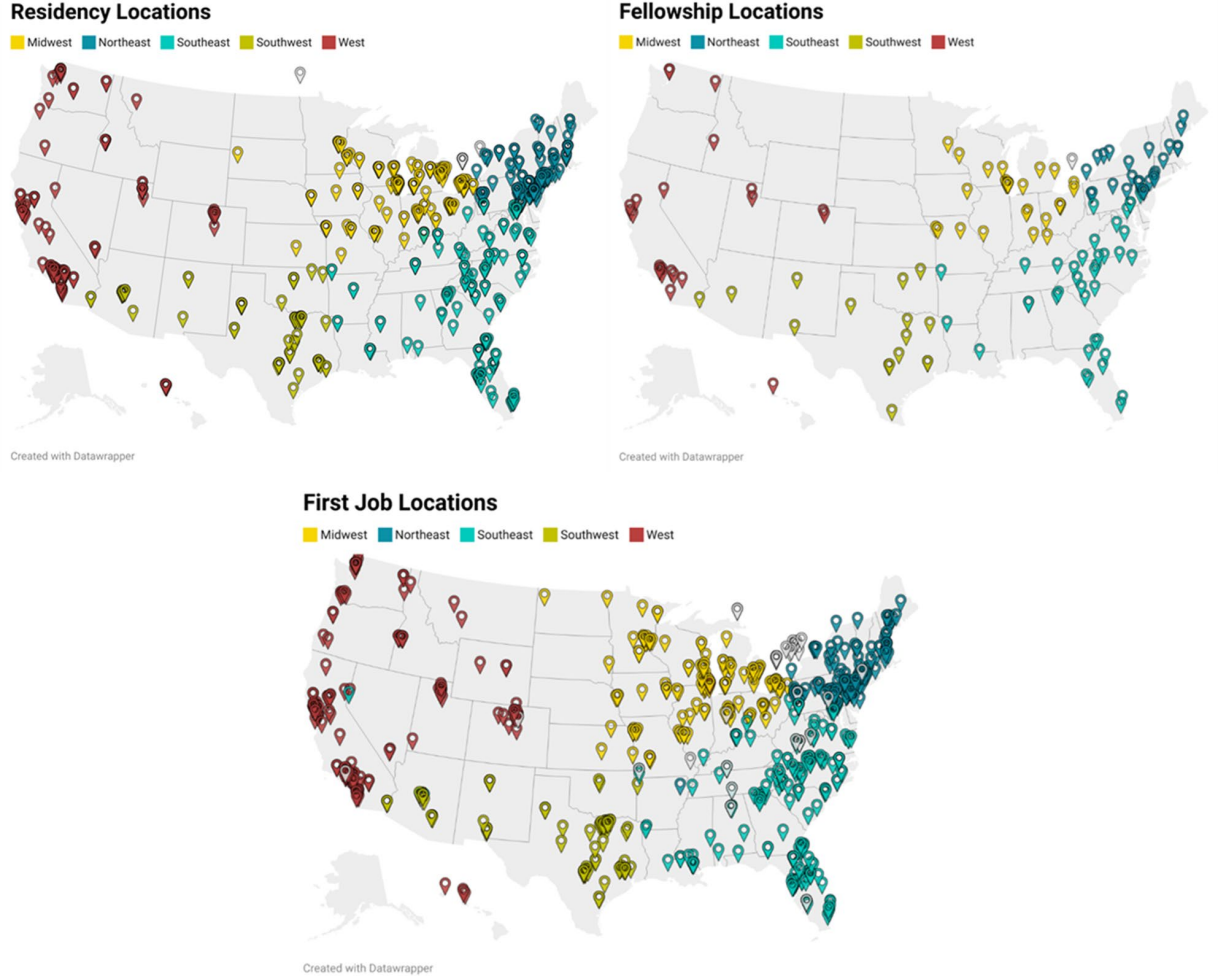
Residency, sports medicine fellowship, and first job pinpoint locations of included fellows

The largest proportion of fellows (25.6%) completed a residency program in the Midwest. Other common locations for residency programs were the Southeast (24.2%), Northeast (22.1%), West (16.4%), and Southwest (10.0%) (Fig. 1).

These trends are echoed in the fellowship and first job location maps and Table 1 as well. Similarly, these locations are illustrated using pinpoint maps, showing the locations where SM fellows completed their fellowship and their first job location after graduation (Fig. 1).

**Table 1:** Residency, Fellowship, and first job location regional distribution

Region	Residency Location (%)	Fellowship Location (%)	First Job Location (%)
Northeast	22.1	19.4	20.3
Southeast	24.4	28.4	24.7
Southwest	10.0	11.9	10.1
Midwest	25.6	22.4	19.1
West	16.4	17.2	23.0
Other	1.5	0.7	2.8

### Relative distances

**Fig. 2:**
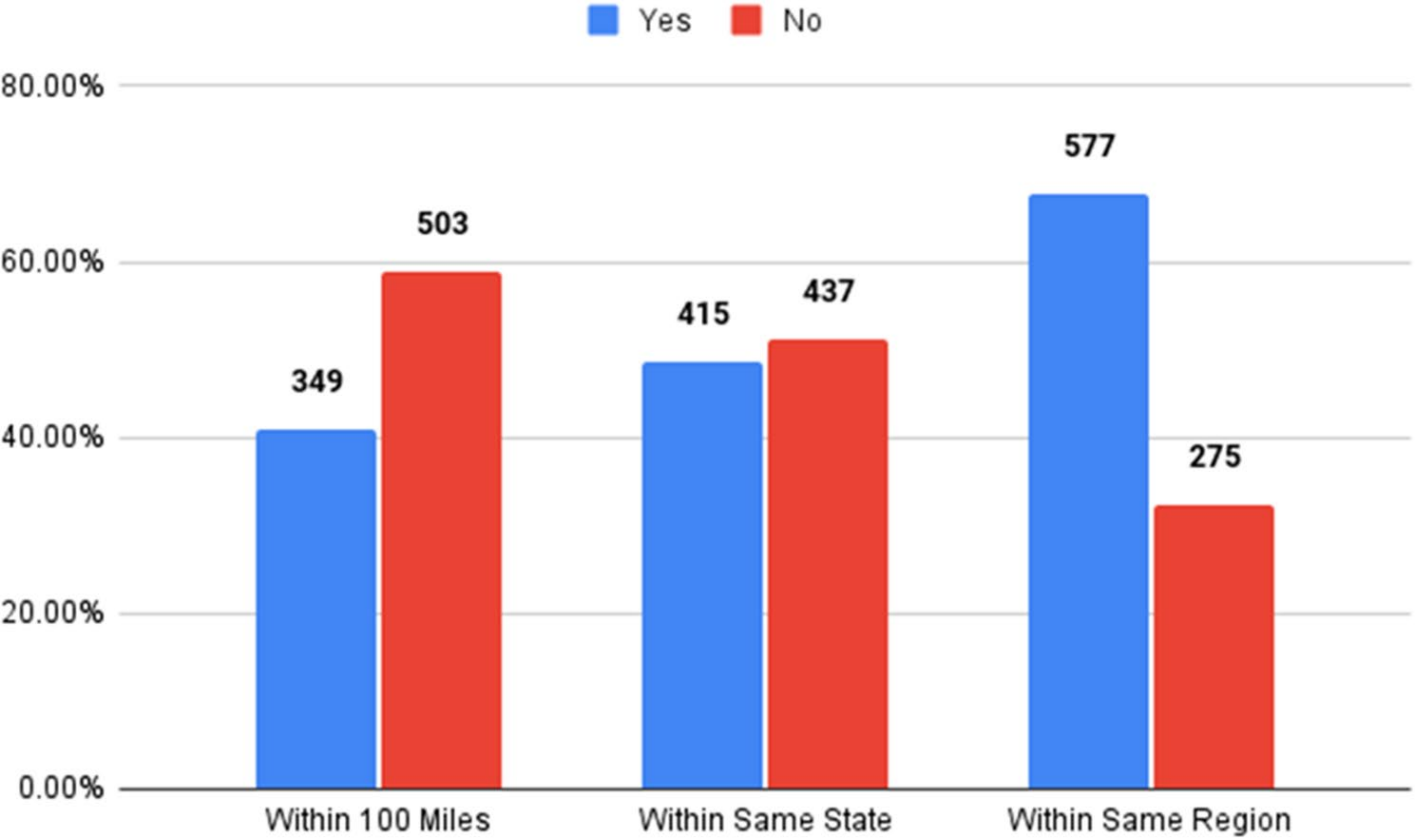
Sports medicine fellowship location

A large proportion of the fellows (40.96%) completed an SM fellowship program within a 100-mile radius of their residency program, 48.71% in the same state, 67.72%within the same region (Fig. 2).

Although the greatest proportion of fellows chose to complete their fellowship within the same region (67.7%) as their residency location, most fellows did not stay within the same state, nor did they complete their fellowship within 100 miles of their residency location. The distribution from residency to fellowship distances was right-skewed. The 10th percentile is 0 miles, the 25th percentile is 2 miles, the 50th percentile is 123 miles, the 75th percentile is 639.8 miles, and the 90th percentile is 1245.5 miles.

Following the SM fellowship, a large proportion of the graduates stayed within 100 miles (41.6%) for their first job, 51.3% stayed within the same state, and 69.9% stayed within the same region. Distribution for fellowship to first job distances was also right-skewed but broader. The 10th percentile was 3.1 miles, the 25th percentile was 19 miles, the 50th percentile was 183 miles, the 75th percentile was 781 miles, and the 90th percentile was 1472.4 miles. These percentiles are listed in Table 2.

**Table 2:** Distance percentiles traveled for fellowship and first job

Percentiles	Residency to Fellowship (miles)	Fellowship to First Job (miles)
Minimum	0	0
10th	0	3.1
25th	2	19
50th	123	183
75th	639.8	781
90th	1245.5	1472.4
Maximum	4693	6503.2

### Specialties before SM

**Fig. 3:**
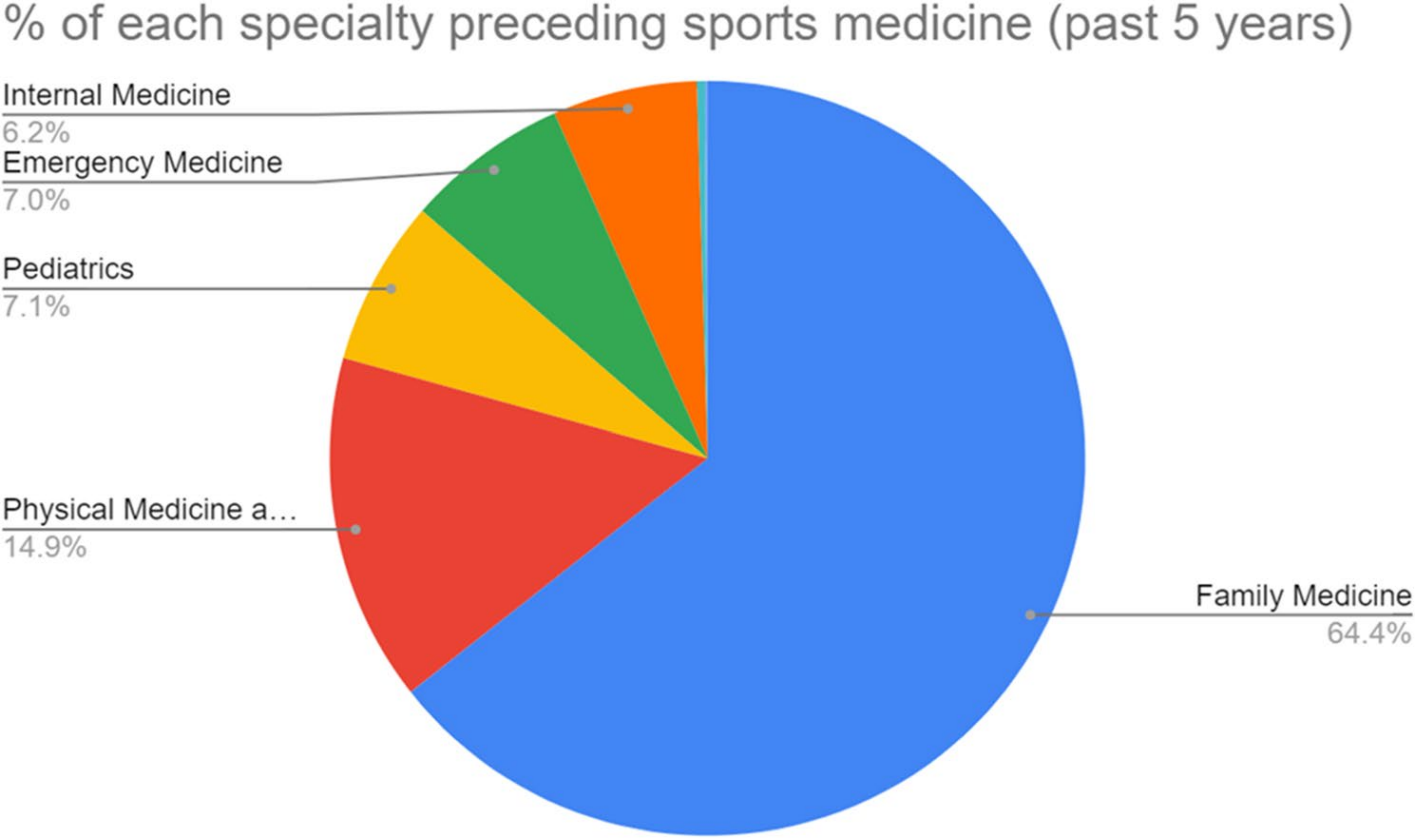
Percentage of residency matching into fellowship

The vast majority of fellows (64.4%) specialized in FM, whereas other fellows had prior training in PM&R (14.9%), Pediatrics (7.1%), EM (7.0%), and IM (6.2%). Three fellows pursued a Neuromusculoskeletal specialty, and one fellow trained in Preventive Medicine. Fig. 3 illustrates the primary residencies that the SM fellows completed.

In all three categories, FM residency graduates were highly likely to stay within 100 miles (OR: 1.47, *p* = 0.009), same state (OR: 1.81, *p* < 0.0001), or same region (OR: 1.61, *p* = 0.002) for their SM fellowship. In contrast, EM residency graduates were more likely not to remain within 100 miles (OR: 0.47, *p* = 0.013) or in the same state (OR: 0.52, *p* = 0.0214). No other significant differences regarding the distance between fellowship and residency location were found among PM&R, Pediatrics, IM, and EM fellows. (Table 3).

**Table 3:** Percentage of PM&R, FM, IM, EM, and pediatrics relative location

Residency	Location of Fellowship Relative to Residency	Percentage Within Location (*n*)	Percentage NOT Within Location (*n*)	OR (95% CI)	*p*-value
Physical Medicine and Rehabilitation	Within 100 Miles	39.2% (49)	60.8% (76)	0.92 (0.62–1.35)	0.695
	Within Same State	41.6% (52)	58.4% (73)	0.71 (0.49–1.04)	0.099
	Within Same Region	64.8% (81)	35.2% (44)	0.86 (0.57–1.27)	0.469
Family Medicine	Within 100 Miles	44.3% (240)	55.7% (302)	1.47 (1.10–1.95)	**0.009**
	Within Same State	54.1% (293)	45.9% (249)	1.81 (1.37–2.42)	**< 0.001**
	Within Same Region	71.6% (388)	28.4% (154)	1.61 (1.20–2.16)	**0.002**
Pediatrics	Within 100 Miles	32.8% (20)	67.2% (41)	0.69 (0.40–1.20)	0.224
	Within Same State	37.7% (23)	62.3% (38)	0.61 (0.36–1.06)	0.084
	Within Same Region	59.0% (36)	41.0% (25)	0.67 (0.39–1.14)	0.155
Internal Medicine	Within 100 Miles	36.2% (17)	63.8% (30)	0.81 (0.45–1.47)	0.544
	Within Same State	40.4% (19)	59.6% (28)	0.70 (0.38–1.27)	0.294
	Within Same Region	57.4% (27)	42.6% (20)	0.63 (0.35–1.14)	0.148
Emergency Medicine	Within 100 Miles	25.4% (15)	74.6% (44)	0.47 (0.25–0.85)	**0.013**
	Within Same State	33.9% (20)	66.1% (39)	0.52 (0.30–0.91)	**0.021**
	Within Same Region	61.0% (36)	39.0% (23)	0.73 (0.43–1.25)	0.252

Bolded p-values are statistically significant

OR (95% CI), Odds Ratio (95% Confidence Interval)

#### First job location

Following their SM fellowship, graduates from an FM background were more likely to stay within 100 miles (OR: 1.44, *p* = 0.016) and within the same state (OR: 1.63, *p* = 0.001). Contrastingly, graduates from a PM&R background were less likely to stay within 100 miles (OR: 0.50, *p* = 0.002) and the same state (OR: 0.48, *p* = 0.0006). No significant differences were found for the other specialties (Table 4).

**Table 4:** Percentage of fellows concerning specialty relative location to first job

Residency	Location of Fellowship Relative to First Job	Percentage Within Location (*n*)	Percentage NOT Within Location (*n*)	OR (95% CI)	*p*-value
Physical Medicine and Rehabilitation	Within 100 Miles	27.9% (31)	72.1% (80)	0.50 (0.32–0.77)	**0.002**
	Within Same State	36.0% (40)	64.0% (71)	0.48 (0.32–0.73)	**0.0006**
	Within Same Region	65.8% (73)	34.2% (38)	0.80 (0.52–1.22)	0.302
Family Medicine	Within 100 Miles	44.7% (230)	55.3% (284)	1.44 (1.10–1.95)	**0.016**
	Within Same State	55.6% (286)	44.4% (228)	1.63 (1.22–2.19)	**0.001**
	Within Same Region	71.6% (368)	28.4% (146)	1.25 (0.91–1.71)	0.168
Pediatrics	Within 100 Miles	35.0% (21)	65.0% (39)	0.74 (0.43–1.28)	0.280
	Within Same State	43.3% (26)	56.7% (34)	0.71 (0.42–1.20)	0.200
	Within Same Region	63.3% (38)	36.7% (22)	0.72 (0.42–1.25)	0.248
Internal Medicine	Within 100 Miles	37.8% (17)	62.2% (28)	0.84 (0.45–1.57)	0.590
	Within Same State	48.9% (22)	51.1% (23)	0.90 (0.49–1.65)	0.737
	Within Same Region	68.9% (31)	31.1% (14)	0.95 (0.50–1.82)	0.875
Emergency Medicine	Within 100 Miles	51.7% (30)	48.3% (28)	1.55 (0.91–2.65)	0.110
	Within Same State	55.2% (32)	44.8% (26)	1.18 (0.69–2.02)	0.543
	Within Same Region	70.7% (41)	29.3% (17)	1.04 (0.58–1.87)	0.897

Bolded p-values are statistically significant

OR (95% CI), Odds Ratio (95% Confidence Interval)

## Discussion

This analysis of SM fellowship program locations compared to residency program locations provides insights into geographic trends notable for medical students, residents, advisors, and program directors. More than half of the fellows stayed within the same geographical region, which illustrates the possibility of a disproportionate spread of fellowships across the United States. Furthermore, this trend was observed with the first job placement after graduation. Most fellows appear to stay within their preferred regions; however, this highlights that regions without programs can remain underserved if trainees in these areas have fewer professional opportunities to stay close by. Geographic regions with more residency programs may have easier access to subsequent fellowships through networking and other opportunities. The Western and Southwestern regions had fewer residency and fellowship programs overall, highlighting the regional variations in training locations [6]. Programs may have incentives to retain fellowship graduates to practice in the same region as well, further contributing to a disproportionate spread of SM physicians. However, relocating to a different region can be attributed to personal factors such as the desire to return home, geographic preferences, limited personal or professional connections to the area, and quality of life considerations.

Personal and family considerations may sway a residency graduate’s preferences for fellowship location, but limited professional factors in certain regions or programs with a curriculum/resources that don’t match up with the residency graduates’ interests also play a significant role. Moving for residency and a subsequent fellowship can make settling down with a family, building community ties, and maintaining social and professional networks more difficult [7]. Recent studies have found that lifestyle factors such as family/vacation time, location, and scheduling become more important as individuals progress through their careers [8–10]. Several other recent studies evaluating factors towards the selection of primary care and surgical residencies likewise place a heavy emphasis on location, with a trend towards staying in the same region or the same institution [11–16]. The location of a residency can be an important predictor of future practice location, as mentioned by the Association of American Medical Colleges (AAMC) in reports available between 2012 and 2021, where more than half (55.2%) of the graduates were found to practice in the same state as their residency program [17]. Thus, this data further demonstrates the increased likelihood of fellows staying within their same geographic region and encourages the creation of more programs in areas that seek more SM physicians. However, it must be acknowledged that although applicants ultimately determine their rank list, the National Resident Matching Program (NRMP) match has an applicant-favored algorithm that assigns positions based on these preferences.

The primary specialty training that SM fellows completed before fellowship was: FM (64.4%), PM&R (14.9%), Pediatrics (7.1%), EM (7%), IM (6.2%), Neuromusculoskeletal medicine (0.3%), and Preventive medicine (0.1%). FM residents may have an advantage when applying for SM fellowships, given their high predominance amongst SM fellows. However, this may be attributed to some SM programs only being available to FM residency graduates [18]. This trend has evolved over the years, with fellowship programs increasing acceptance and consideration for more specialties. Due to the multidisciplinary aspects of SM, fellowship programs will ideally continue to accept and promote applicants with residency training in various disciplines [1].

As this study has found, there is a trend for fellows to remain within the same geographical region as their residency. Although our study did not directly evaluate healthcare disparities, previous literature has shown that rural areas do not have the same level of access to healthcare services and specialists compared to urban areas [12]. One factor that can affect geographical inequity is student background, as students from rural backgrounds or who have experience in a rural community have a higher likelihood of practicing in a rural community [12]. A similar trend is observed for students from an urban background who are more likely to remain in urban areas. A majority of SM fellowships are concentrated in large cities [6]. Thus, health equity is an issue due to the unequal distribution of providers. This encourages programs to recruit qualified individuals from rural backgrounds and promote more exposure for students in different environments [19]. Fellows from rural backgrounds can be a proposed strategy, as these individuals may be more likely to return to and practice in rural areas. Specialty choices ultimately have profound effects on healthcare delivery, as seen through the imbalance between the supply of physicians in particular specialties and demand in patient care, especially in rural areas. Geographical inequality can lead to reduced access to physicians, resulting in a ripple effect towards unsatisfactory patient outcomes and reduced health equity. SM has evolved from centering around athletes to encompassing the general population. As a result, the patient population in need of SM is everywhere. As advocacy of physical activity has increased over the years to combat risk for chronic diseases, the demand for SM physicians will also continue to increase, necessitating the need for equal distribution of physicians [20].

### Implications

Understanding these geographic trends has implications for both applicants and programs. This is especially pertinent information as most primary care SM websites do not provide comprehensive program information [21]. Location is a major consideration during the application process for both medical students and residents, especially if they have certain long-term goals in mind. Applicants can place more emphasis on particular residency programs if they have a specific fellowship program in mind. The proximity of fellowship programs can be a deciding factor for students when choosing a specific residency and acts as a guide for future medical students and residents when considering program selections. Fellowship training can require a fairly quick relocation, and understanding the national trends can help applicants plan out personal, financial, and logistical factors. For fellowship programs, geographic proximity patterns provide insight into where the applicant pool tends to originate from, which can support targeted recruitment strategies. On a broader scale, analyzing how physicians relocate throughout different stages of training can help provide context for workforce distribution trends and identify areas that can benefit from targeted training expansion. Although these regional variations are notable, we did not have data on population size, actual SM physician distribution, or regional SM needs. Thus, our analysis is strictly limited to describing the geographic patterns.

### Limitations

Our study provides a general understanding of the locations that residents matched into for SM fellowships over the past five years, but it must be considered within the context of its limitations. First, we selected this time range for data collection as it provides the most recent trends and data, but is limited by the range of the total available data. This also limits the generalizability as it includes both pre- and post-pandemic data. Future studies would be needed to validate whether these trends persist post-pandemic. Second, we were unable to obtain data from all programs listed in the American Medical Society for Sports Medicine (AMSSM) directory, with programs either not responding or declining to participate, thus rendering our results susceptible to non-responder bias. Although the programs included were generally evenly spread, there was a regional variation, with more responses from programs in the West and Southeast, and fewer from the Southwest. This can limit the generalizability of our region-specific trends.

Additionally, applicant-level geographic data was not available, which prevents comparison of the geographical diversity of applicants with that of the matched fellows. Thus, the determination of the cause of the observed regional retention could not be ascertained due to program selection or applicant preferences. Third, we did not analyze the number of fellowships that only accepted graduates from specific primary residencies. Therefore, the predominance of FM residency graduates in our study may be due to fewer programs admitting graduates from other specialties, but also mirrors national patterns in which SM fellowship positions are mainly filled by FM applicants. Furthermore, residency programs are not evenly distributed across the primary residencies evaluated here. For example. FM has substantially more programs nationwide compared to EM and PM&R. The greater program density can increase geographic proximity between fellowships and subsequent first job opportunities. This can also influence the geographic trends, as FM applicants may have more flexibility to apply regionally compared to other specialties. Thus, the geographic clustering found in this study can reflect fellowship selection trends, the underlying baseline characteristics of the applicant pool, and the training infrastructure rather than causal specialty effects. Finally, our data collection focused primarily on objective data on the relative location between residency and SM fellowship. The factors and motives associated with the fellows’ choices were not collected. SM workforce density, regional SM healthcare needs, and population size were not assessed as well, which prevents the determination of whether these geographic patterns are leading to geographical inequity. Future studies in this field can be conducted by analyzing the specific factors, motives, and rationale that influence residency graduates’ choice of a particular fellowship. This qualitative information can be elicited through surveys or personal interviews, but will be insightful in understanding the different factors that influence a residency graduate’s choice of fellowship, not only in SM but also compared to other specialties.

## Conclusion

Our study concludes that residency graduates are more likely to remain in the same geographic region for their SM fellowship. FM had the highest continuity close to their training location as well as their first job, while EM had the lowest likelihood of staying close by. Interestingly, for PM&R, these graduates had a low probability of staying in the area for their first job. This data provides insights into the geographic mobility of recent SM fellows for interested applicants. Understanding these trends allows applicants to set expectations, anticipate relocation, and make informed choices when planning their potential training pathways.

## Data Availability

The datasets used and/or analyzed in the current study are available upon reasonable request. Please contact J.W. to request data from the study.

## Abbreviations

SM: Sports medicine
PM&R: Physical Medicine & Rehabilitation
FM: Family medicine
IM: Internal medicine
EM: Emergency medicine
ACSM: American College of Sports Medicine
ACGME: Accreditation Council for Graduate Medical Education
IRB: Institutional Review Board
OR: odds ratio
AAMC: Association of American Medical Colleges
NRMP: National Resident Matching Program
AMSSM: American Medical Society for Sports Medicine
